# Diffuse Cutaneous Mastocytosis: A Current Understanding of a Rare Disease

**DOI:** 10.3390/ijms25031401

**Published:** 2024-01-23

**Authors:** Agnieszka Rydz, Magdalena Lange, Hanna Ługowska-Umer, Monika Sikorska, Roman J. Nowicki, Cristina Morales-Cabeza, Iván Alvarez-Twose

**Affiliations:** 1 Student’s Scientific Circle Practical and Experimental Dermatology, Medical University of Gdansk, 80-211 Gdańsk, Poland; agnieszka.rydz@gumed.edu.pl; 2 Department of Dermatology, Venereology and Allergology, Medical University of Gdańsk, 80-211 Gdańsk, Poland; hanna.lugowska-umer@gumed.edu.pl (H.Ł.-U.); msikorska@gumed.edu.pl (M.S.); rnowicki@gumed.edu.pl (R.J.N.); 3 Instituto de Estudios de Mastocitosis de Castilla-La Mancha (CLMast)—Spanish Reference Center for Mastocytosis, Hospital Virgen del Valle—Complejo Hospitalario Universitario de Toledo, 45071 Toledo, Spain; cmcabeza@sescam.jccm.es (C.M.-C.); ivana@sescam.jccm.es (I.A.-T.)

**Keywords:** diffuse cutaneous mastocytosis, pediatric mastocytosis, diagnosis, treatment, tryptase

## Abstract

Mastocytosis is a heterogeneous disease characterized by the expansion and accumulation of neoplastic mast cells in various tissues. Diffuse cutaneous mastocytosis (DCM) is a rare and most severe form of cutaneous mastocytosis, which typically occurs in childhood. There have been reports of a familial DCM with specific gene mutations, indicating both sporadic and hereditary factors involved in its pathogenesis. DCM is associated with severe MC mediator-related symptoms and an increased risk of anaphylaxis. The diagnosis is based on the appearance of skin lesions, which typically show generalized thickening, erythroderma, blistering dermographism, and a positive Darier’s sign. Recognition, particularly in infants, is challenging due to DCMs resemblance to other bullous skin disorders. Therefore, in unclear cases, a skin biopsy is crucial. Treatment focuses on symptom management, mainly including antihistamines and mast cell stabilizers. In extremely severe cases, systemic steroids, tyrosine kinase inhibitors, phototherapy, or omalizumab may be considered. Patients should be equipped with an adrenaline autoinjector. Herein, we conducted a comprehensive review of literature data on DCM since 1962, which could help to better understand both the management and prognosis of DCM, which depends on the severity of skin lesions, intensity of mediator-related symptoms, presence of anaphylaxis, and treatment response.

## 1. Introduction

Mastocytosis is a rare condition characterized by an abnormal accumulation of neoplastic mast cells (MCs) in various tissues, mostly including the skin, bone marrow (BM), spleen, liver, gastrointestinal tract, and lymph nodes [1,2,3]. In 2016, the World Health Organization (WHO) categorized the disease into three primary clinical variants: cutaneous mastocytosis (CM), systemic mastocytosis (SM), and the locally aggressive disease known as MC sarcoma (MCS) [4]. The current updated classification of mastocytosis is presented in Table 1 [1,2,3,4,5].

In contrast to adults, pediatric patients usually suffer from CM and only rarely from SM [6,7,8]. CM is diagnosed on the basis of the typical morphology of skin lesions and the absence of signs or criteria of SM [9]. According to the EU/US consensus group, the diagnostic criteria of CM include the presence of typical skin lesions of mastocytosis together with the Darier’s sign, which is a major CM criterion, and one or two of the minor criteria—increased numbers of MCs in biopsy sections of lesional skin and an activating *KIT* mutation at codon 816 in lesional skin [6,9]. The Darier’s sign is characterized by reddening and urticarial swelling of the lesion after its mechanical irritation [6,8]. CM is subdivided into three main subtypes: the most common form, namely maculopapular CM (MPCM, also referred to as urticaria pigmentosa, with two variants—monomorphic MPCM and polymorphic MPCM); mastocytoma of the skin; and diffuse CM (DCM); which is the least common form of the disease [6,7,8,10,11].

DCM represents the most severe clinical manifestation of CM and is characterized by an extensive infiltration of MCs throughout the entire skin in the absence of macroscopic individualized cutaneous lesions [6,8]. This condition is typically observed in early childhood and may persist into adulthood in some cases [8]. The frequency of DCM ranges from 2% to 11% of all forms of pediatric CM [10,11,12,13,14,15,16]. In the largest systemic review of 1747 cases of pediatric mastocytosis, skin involvement presenting as DCM included 5.2% of patients [7].

The pathogenesis of mastocytosis is closely associated with gain-of-function somatic mutations in the *KIT* gene, resulting in the steam cell factor (SCF)-independent activation and phosphorylation of the KIT receptor, which drives differentiation, survival, and accumulation of MCs in various organs [1,17,18]. A *KIT* mutation involving codon 816 in exon 17 (most commonly the *KIT* D816V mutation) is found in the majority of adult patients with SM [1,17]. In contrast, pediatric patients, who mostly present with CM, may exhibit a mutation of codon 816 in exon 17 in approximately 42% (including the *KIT* D816V mutation in 36%), as well as different somatic or germline *KIT* mutations, mainly in exons 8, 9, and 11 (approximately 44%), or have no *KIT* mutation (wild-type genotype, approximately 14%) [16,18]. The clinical presentation of mastocytosis is related to the release of MC mediators, which results in a variety of clinical symptoms, ranging from mild symptoms such as flushing, pruritus, dyspnea, abdominal pain, vomiting, and diarrhea to potentially life-threatening conditions such as hypotension and anaphylactic shock [6,10,11,19]. Upon activation, MCs promptly initiate degranulation, leading to the release of preformed mediators stored in cytoplasmic granules, including histamine, serotonin, heparin, chymase, chondroitin sulfate, carboxypeptidase, tryptase, and TNF-α [20]. This initial phase is followed by a de novo synthesis of membrane lipid-derived mediators, principally prostaglandin D2 (PGD2), cysteinyl leukotrienes (LTC4, D4, and E4), and platelet-activating factor (PAF). As MC activation continues, a set of cytokines, both pro-inflammatory and anti-inflammatory, is also newly synthesized. This includes TNF-α, GM-CSF, IL-1, IL-3, IL-4, IL-5, IL-6, IL-13, IL-1RA, chemokines such as IL-8, CCL-2, CC-3, CCL-5, and CXCL-8, along with growth factors like transforming growth factor-beta 1 (TGF-β1), SCF, fibroblast growth factor (FGF), nerve growth factor (NGF), platelet-derived growth factor (PDGF), vascular endothelial growth factor (VEGF), and interferons [20,21]. In recent years, the role of some cytokines and chemokines in inducing MC mediator-related symptoms has been examined in humans with mastocytosis [22,23,24,25,26,27]. It has been found that IL-6 may act as an autocrine growth factor for neoplastic MCs; IL-31 is associated with itching of the skin; and oncostatin-M and monocyte chemoattractant protein-1 (MCP-1/CCL2) impact BM remodeling and modulate the BM microenvironment [22,23,24,26,27]. Moreover, it has been shown that blistering within the lamina lucida (junctional) is attributed to serine proteases released from MCs, which infiltrate the upper dermis [28]. Activation of the MCs in patients with mastocytosis leads to the elicitation of multiple mediators, which may be provoked by numerous triggers [20,29]. Most often, MCs can be activated by Immunoglobulin E (IgE)-mediated mechanisms (such as Hymenoptera venoms, plants, food, and some drugs, e.g., nonsteroidal anti-inflammatory drugs [NSAIDs], quinolones, neuromuscular blocking agents [NMBAs], and radio-contrast media [RCM], among others). Non-IgE-mediated mechanisms include activation of the complement system (e.g., C5aReceptor/CD88 mediate a reaction to polyethyleneglycol [PEG] and polysorbate included in vaccines), direct MCs degranulation mediated by MAS-related G Protein-Coupled Receptor-X2 (MRGPRX2) activated by neuropeptides and some drugs (e.g., opiates, quinolones, vancomycin, NMBAs, RCM), activation pathways through Toll-like receptors (involved in response to bacteria, parasites, viruses), and stimulation by physical factors (heat, cold, pressure, stress, physical exertion, the friction of mastocytosis skin lesions), among others [20,30,31,32,33]. However, in a subset of patients, the mechanism and triggers of MC activation and anaphylaxis remain unknown. In contrast to adults in whom hymenoptera venom is the most common trigger of MC activation, most reported cases of children with mastocytosis who suffer from anaphylaxis are idiopathic or induced by drugs and food [12,29,30,34].

## 2. Genetic Background of Diffuse Cutaneous Mastocytosis

According to current knowledge, genetic drivers, epigenetics, and both hormonal and metabolic factors involved in the pathogenesis of pediatric mastocytosis are poorly understood [1,3,8]. However, numerous studies show that children with DCM can carry different somatic mutations in exon 17 of *KIT* (D816V, D816Y, or D816I), as well as mutations in other exons (Del419, K509I, or internal tandem duplication A502_Y503dup) [18,35,36,37,38,39,40,41]. Familial mastocytosis is a specific form of the disease in which mostly germline *KIT* mutations are detected in affected family members [42,43,44,45,46,47,48,49,50]. Interestingly, familial mastocytosis associated with deafness has also been reported [51]. Familial DCM has been reported in three families in which germline mutations, including the A533D and the p.S451C *KIT* mutations, were found [52,53,54]. Additionally, rare cases of DCM associated with gastrointestinal stromal tumors (GIST) and tuberous sclerosis have been described [36,49,50,55,56]. It has been found that patients with DCM and coexisting GIST systematically show germline *KIT* mutations such as S476I and Del419Asp [6,49,55]. Of note, some children with DCM may suffer from a biological variant of SM known as well-differentiated SM (WDSM), in which BM MCs have a mature, apparently normal morphology, usually lacking CD25 and CD2 expression, and often display an aberrant expression of CD30 in the absence of *KIT* codon 816 mutations [56,57]. Interestingly, the K509I *KIT* mutation located at exon 9 of the gene has been reported in some patients with WDSM presenting as DCM [36,56]. Altogether, these findings reflect the complex genetic background of DCM and highlight a potential hereditary component in a significant fraction of cases.

## 3. Clinical Presentation of Diffuse Cutaneous Mastocytosis

**Figure 1:**
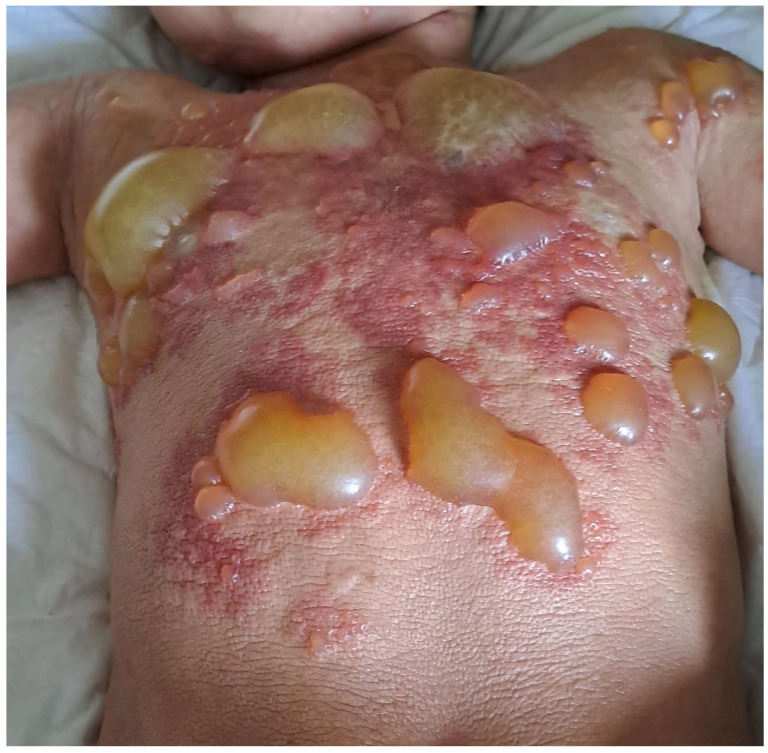
DCM with extensive blistering in an infant.

DCM manifests as generalized erythema, usually with pachydermia (thickened skin), associated with pronounced dermographism or a positive Darier’s sign, which are evident after minimal mechanical irritation of the skin [6]. Extensive blistering is a typical feature of DCM in the infantile period [6,19,40,58,59] (Figure 1). It is worth pointing out here that the diagnosis of DCM should be established only in children with generalized thickened and darker than normal skin, but not in those with extensive, confluent MPCM or bullous lesions [6]. The current classification of CM does not distinguish bullous mastocytosis as a separate form of the disease because blistering may also occur in MPCM and mastocytoma [6]. In infants, DCM may present with large hemorrhagic blisters and with small vesicular lesions; however, both variants may coexist [8,19,59]. The hemorrhagic nature of bullous lesions, as well as prolonged bleeding from skin wounds in some children, may be related to the local release of heparin from dermal MCs [6]. Papules can be present in pachydermatous skin areas. Occasionally, DCM may present as generalized erythema with pseudoxanthomatous or tumor-like lesions [6,11,59,60]. Clinical manifestations of DCM tend to evolve with age [6,8]. Extensive blistering is predominantly observed in infancy and usually ceases within 2 or 3 years of age (Figure 1), while diffuse pachyderma with slight brown or yellow discoloration and a leather-like skin appearance develop with time. Children with DCM usually present with numerous symptoms associated with the release of MC mediators, such as itching, flushing, hypotension, headache, abdominal cramping, or diarrhea [13,14,28,40,58,61]. It has also been found that baseline serum tryptase levels are significantly higher in patients with DCM than in patients with other forms of CM [10,13,14]. Moreover, children with DCM are at a higher risk of developing severe MC mediator-related symptoms, such as sudden hypotension or anaphylactic shock [10,13,59]. In a study analyzing 10 pediatric patients with DCM, three episodes of anaphylaxis were observed in patients with basal tryptase levels of 22, 103, and 2.7 ng/mL, respectively. These severe reactions were provoked by clindamycin, ketamine, magnetic resonance imaging-contrast medium, and an unidentified trigger [59]. In other studies, anaphylaxis in children with DCM was provoked by an unknown factor, either after a meal or during a peripheral intravenous placement [52,62,63]. The presence of severe MC mediator-related symptoms in children with DCM may be primarily attributed to the extensive and substantial infiltration of MCs throughout the skin. Furthermore, numerous studies have shown that rubbing and scratching of the skin, sudden temperature changes (such as hot baths or exposure to heat), teething, viral infections, or vaccinations may induce blistering or provoke other MC mediator-related symptoms [13,28,40,58,59]. A brief review of case reports on DCM, published since 1962, is presented in Table 2 [28,35,36,38,39,40,41,52,53,54,58,60,62,63,64,65,66,67,68,69,70,71,72,73,74,75,76,77,78,79,80,81].

As mentioned above, the familial occurrence of DCM has also been reported [52,53,54]. Interestingly, five patients with DCM in three generations within a single family have been described [53]. In all these cases, the symptoms started during infancy and initially presented as diffuse thickening of the skin, blistering, pruritus, and dermographism. DNA sequencing revealed a germline mutation in the transmembrane domain of the *KIT* gene (the A533D mutation) in all five family members [53]. Another report of familial DCM concerned a 35-year-old man who was suffering from generalized bullous skin lesions until the age of 2 and his 8-year-old son, who experienced nearly identical symptoms [54]. In both cases, genetic examination revealed a germline mutation in the p.S451C domain of the *KIT* gene [54]. Recently, another familial DCM, associated with germline *KIT* A533D (an autosomal dominant gain-of-function germline *KIT* variant: c.1598C > A, p.Ala533Asp), has been reported in a six-month-old boy with bullous lesions and fever and in his mother, who had a history of blistering in childhood and carried the same mutation [52].

## 4. Diagnostics and Differential Diagnosis

**Figure 2:**
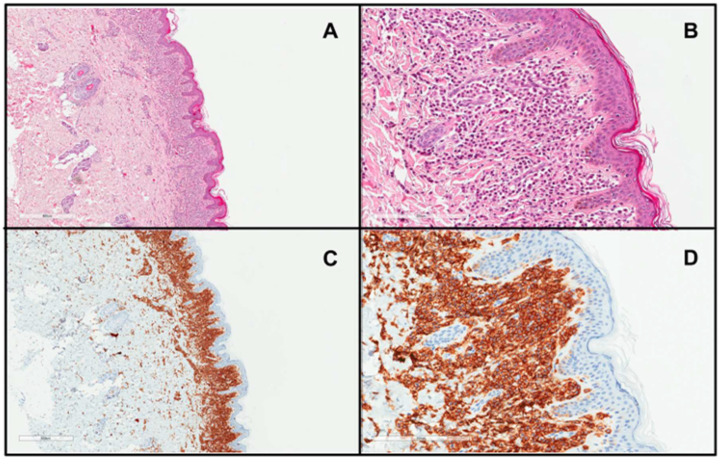
Skin biopsy from a patient with DCM showing a marked infiltrate of round to polygonal MCs occupying the whole papillary dermis ((**A**), H&E stain ×4; (**B**), H&E stain ×20; (**C**), CD117 stain ×4; (**D**), CD117 stain ×20)).

The diagnosis of DCM is mainly based on the clinical presentation, which includes thickening of the entire skin, erythroderma with blistering, prominent dermographism, or a positive Darier’s sign usually associated with the presence of MC mediator-related symptoms [6,8,61]. In children with DCM, the Darier’s sign should be elicited with caution, particularly in infants, because hard stroking of the skin may lead to flushing or hypotension due to massive MC degranulation [6,28,82]. In unclear cases, lesional skin biopsy and immunohistochemistry should be conducted with antibodies against tryptase and/or CD117, which is the gold-standard [6,8,83] (Figure 2). MC infiltration in the dermis (Figure 2) may be accompanied by subepidermal edema, causing vesiculobullous lesions [28,58]. Moreover, molecular analysis of *KIT* mutations in lesional skin may be used to confirm the diagnosis of CM [7]. The determination of baseline serum tryptase levels is also considered a valuable tool for diagnosing and monitoring DCM patients [8,61,83]. However, it is important to note that elevated tryptase levels can strongly suggest a diagnosis of mastocytosis but do not conclusively confirm it. There are many other conditions presenting with elevated basal serum tryptase levels, such as hereditary alpha-tryptasemia, allergies, chronic eosinophilic leukemia, and some nephropathies [20,82]. It is worth pointing out here that children with DCM commonly have elevated serum tryptase levels even in the absence of an underlying SM in many cases, which is due to the extensive MC burden in the entire skin [14,15,28,59]. In all DCM children, a physical examination including inspection of the skin, abdominal palpation, abdominal ultrasound, serum chemistry, and a complete blood count with differential are also recommended [8,83]. Further diagnostic evaluations, including BM studies, are indicated only in selected cases with highly suspected systemic involvement and are not universally recommended [8,19,59,61,83].

DCM, particularly in the infantile period, often represents a diagnostic challenge due to a wide spectrum of diseases that may resemble DCM, encompassing mainly staphylococcal scalded skin syndrome (SSSS), epidermolysis bullosa (EB), impetigo bullosa (IB), erythema multiforme (EM), atopic dermatitis, Langerhans cell histiocytosis, linear IgA bullous dermatosis, and incontinentia pigmenti [28,59,69,76,81,84,85,86,87,88]. It is widely believed that misdiagnosis of DCM can be attributed to at least two factors: the absence of maculopapular or plaque lesions, which are most typical for CM, and the rare occurrence of DCM [59]. Table 3 provides a brief summary of the clinical characteristics of diseases that should be considered in the differential diagnosis of DCM in children [28,59,69,76,81,84,85,86,87,88].

## 5. Treatment

Treatment of DCM predominantly focuses on trigger avoidance and the management of MC mediator-related symptoms [8,40,58,83]. Parents and caregivers should be informed that friction, rubbing, heat exposure, sudden temperature changes, teething, fever, and vaccines may provoke exacerbations of skin lesions, blistering, or anaphylaxis [8,30,58,82,89,90]. Avoidance of skin irritation is one of the most important rules in the skin care of children with DCM.

### 5.1. Topical Therapy

In children with blistering and denuded skin areas, topical antibiotics or antiseptics are indicated to prevent skin infections [8,30]. Mupirocine ointment or fucidic acid creams are commonly used [30]. To reduce blistering and pruritus, topical mild- or medium-potency corticosteroids in short-term therapy may be considered [41,81,83]. Therapy with mometasone furoate 0.1% cream applied once daily to the lesions of DCM infants showed essential improvement in erythema and a decrease in bullae formation [40]. However, topical corticosteroids should be used cautiously, in short-term therapy, and for limited skin areas to avoid side effects, particularly skin atrophy and adrenal suppression therapy [30,41,81]. Supportive topical treatment, which includes creams containing disodium cromoglycate (0.2% to 4%), should only be used for intact skin [41,81,91].

### 5.2. Oral Therapy

First-line therapy includes antihistamines, which block H_1_ receptors, and MC stabilizers [8,30,83,92]. Flushing, blistering, and itching may be reduced mainly by using second-generation antihistamines (cetirizine, loratadine, desloratadine, fexofenadine, levocetirizine, rupatadine, and bilastine) and MC stabilizers (e.g., ketotifen and sodium cromoglycate) [8,77]. First-generation antihistamines (chlorpheniramine, diphenhydramine, hydroxyzine, and azelastine) are also used in children, mostly in those with very severe pruritus, as they reduce itching more effectively than second-generation antihistamines [30,92]. In unresponsive cases, second-generation antihistamines’ dose can be increased up to four times the standard dose for age [8,30,92]. To reduce gastrointestinal symptoms, H_2_ antagonists, proton pump inhibitors, and oral MC stabilizers are recommended [8,30,58,83,93]. If MC mediator-related symptoms persist, an add-on of antileukotrienes may be considered [92]. In severe blistering unresponsive to standard antimediator therapy (H_1_-antihistamines, cromolyn sodium, H_2_-antihistamines, proton pump inhibitors, and leukotriene antagonists), oral steroids in short-term therapy may be applied [8,30]. Oral steroids are very effective, but in long-term use, are associated with serious side effects like growth retardation, skin changes, muscle weakness, and obesity, among others [83,92].

As patients with DCM are at a higher risk of anaphylaxis, adequate training on adrenaline autoinjector (epinephrine) administration is crucial [30]. A typical dosage of adrenaline in children is 0.01 mg/kg [30,94]. In Europe, prefilled autoinjectors are available, offering a dose of 0.15 mg for children ranging from 7.5 to 30 kg, with variations based on the autoinjector’s license [30,94]. Whenever needed (e.g., anaphylaxis), adrenaline should be administered via intramuscular injection into the mid-outer thigh and, if necessary, can be repeated every 5 to 15 min (with the maximum dosage being 0.5 mg) [94]. In the event of cardiovascular or respiratory reactions, accompanying measures include the administration of high-flow oxygen, patient positioning (e.g., Trendelenburg position with elevation of the lower limbs for improving/preventing hypotension), and the use of inhaled adrenaline or beta-agonists such as salbutamol [94]. Tyrosine kinase inhibitors may be considered only in life-threatening cases; generally, they are reserved for SM [8,58,61,83]. A successful treatment with imatinib has been reported in two infants with DCM carrying an exon 8 *KIT* mutation (Del419) who underwent antimediator treatment, which failed to prevent severe relapses [95]. In both cases, imatinib treatment was started with an initial dosage of 200 mg/day and resulted in rapid improvement and eventual remission. Therefore, imatinib was gradually tapered and discontinued, with no relapses observed during the 6-month follow-up period [95]. Moreover, successful therapy with imatinib was achieved in three members of a family (a father and two daughters) who were diagnosed with WDSM associated with the K509I germline *KIT* mutation. Two of these patients (the father and one daughter) fulfilled WHO diagnostic criteria for MCL, while the remaining daughter had ISM. In addition, all three patients presented with DCM and showed concomitant GIST; noteworthy, imatinib rapidly induced a complete remission of mastocytosis in all three cases [57]. Similarly, an additional few WDSM cases presenting with DCM showing a complete or near-complete response to imatinib have been reported in the last decade [45,48]. Another therapeutic option for mastocytosis patients, in whom the disease is associated with severe MC mediator-related symptoms and anaphylaxis, may be omalizumab, a monoclonal anti-IgE antibody [96]. Up until now, treatment with omalizumab was reported only in one patient with DCM in whom the therapy was proven to be effective and safe [96]. This patient received monthly subcutaneous injections of 150 mg of omalizumab for three months and remained asymptomatic within the first month following treatment initiation [96].

### 5.3. Phototherapy

In selected cases of CM with severe, recurrent, or persistent MC-mediator release symptoms, refractory to standard antimediator therapy, UVA1 or narrow-band (NB)-UVB phototherapy may be considered, as these therapies have fewer side effects than photochemotherapy (PUVA) and UVA1 has the ability to reach deeper layers of the skin [58,72,97]. PUVA is generally not recommended in children with CM due to the risk of potent side effects of this therapy (mainly the risk of skin cancers, melanoma, cataracts, and hepatotoxicity of psoralen) and the tendency to spontaneously regression of skin lesions around puberty [8].

## 6. Discussion

The rare occurrence of DCM results in a lack of large cohorts of patients, which significantly limits the experience in the diagnosis and management of this rare and severe form of CM, even among the most reference or excellence centers for mastocytosis. In a recent large cohort French study on pediatric mastocytosis, DCM was reported in 15 (5.5%) of 272 children; noteworthy, mastocytosis was congenital in more than half of the cases, but there were no cases of familial mastocytosis [14]. The majority (87%) of these children had MC mediator-related symptoms, and the mean baseline serum tryptase was 23.99 ng/mL (range: 2–60 ng/mL). In a Polish study of 102 patients with childhood-onset mastocytosis, 7 children (6.9%) had DCM, 6 of them presented with blistering, and all had MC mediator-related symptoms, as well as basal serum tryptase over 20 ng/mL [12]. Generally, these results are in line with the data reported by other centers [13,15,19].

Much more diverse are the data on the risk of anaphylaxis among children with DCM, which has been reported with a frequency ranging from 0% to 50% [10,12,19,29,59,98] commonly without a known triggering factor [29,52,59,62]. Importantly, there was no anaphylaxis provoked by vaccination in any of the 13 children with DCM diagnosed by the National Institute of Health in the US [89]; in contrast, one child with DCM from Italy had bullous lesions and bronchospasm after a hexavalent vaccine (diphtheria, tetanus, pertussis, inactivated poliovirus, Haemophilus influenza type B, hepatitis B) [90].

Another issue that deserves discussion is the clinical course of DCM in children. Although some studies have shown no evidence of a complete regression in children with DCM [99], others have revealed an overall rate of regression (complete and partial) of up to 94% [7]. Moreover, a spontaneous decrease in skin involvement has been recently reported in 5 of 12 patients with DCM [15]. Interestingly, this study also showed that a decrease in both skin involvement and serum tryptase levels was systematically observed in patients with sporadic DCM, whereas those with familial DCM displayed no decrease in cutaneous lesions and stable serum tryptase levels [15]. The detailed analysis of the tendency to spontaneous remission in 7 children with DCM who were followed-up for 10 years performed by the Polish group shows that extension, elevation of skin lesions, blistering, and serum tryptase level decreased significantly with time [100]. However, none of these patients experienced a complete or major regression of skin lesions; 6 of 7 patients had a partial regression and 1 exhibited no regression. A complete regression of MC mediator-related symptoms was reported only in one child. The tendency to spontaneous regression was lower in children with DCM than in those with MPCM [100]. Altogether, these findings suggest that spontaneous remission occurs in a significant number of children with DCM, but not in all.

Another important point for discussion concerns the assessment of the true frequency of an underlying SM in patients who present with skin lesions corresponding to DCM. In three series of 15, 10, and 8 children with DCM, respectively, none of them developed SM [14,19,59]. Results of a long-term follow-up of pediatric mastocytosis show that none of the 15 patients with childhood-onset DCM progressed to SM for at least 8 years of the disease duration [14]. However, it has also been shown that children with DCM-like skin lesions may suffer from both WDSM and aggressive SM (ASM) [14,56]. In a French population of children with mastocytosis, 3 of 610 patients had congenital ASM with the *KIT* D816V mutation, and all of them presented with skin lesions corresponding to DCM [14]. Also, a few case studies present children in whom the initial diagnosis was DCM, but further diagnostic procedures revealed SM [68,71,101,102,103,104]. In some children with DCM, a progression to SM over time was also observed [11,13,19,37]. Despite the intensive treatment and fatal outcome of DCM due to the severe course of the disease, infectious complications or comorbidities have been occasionally reported [35,39,41,64,69,75]. Taking all of the above into consideration, the long-term prognosis is good for the majority of children with DCM. Nevertheless, one should keep in mind that children with this form of CM are at risk of severe, life-threatening MC mediator-related symptoms, infections due to extensive blistering, and may develop SM. Therefore, a multidisciplinary approach is highly recommended.

## 7. Conclusions and Future Perspectives

The rare occurrence of DCM and fragmented and sometimes incompatible data on the incidence, symptomatology, and evolution of this disorder indicate the need for collecting data in various centers using a uniform classification, terminology, and grading system for assessing MC mediator-related symptoms and response/spontaneous regression criteria (complete, major, partial, or no regression) depending on the percentage of improvement [6,9]. It is crucial for the analysis of data obtained from distinct countries to appropriately interpret the tendency towards spontaneous regression and the assessment of treatment response. Currently, the standard method of documenting skin involvement is photography. In the future, 3D total body photography may be applied for assessing skin lesions in patients with CM in a more precise way [105]. Moreover, new diagnostic tools, such as reflectance confocal microscopy and two-photon fluorescence lifetime imaging, may become useful in children in whom non-invasive procedures are of major importance [106,107].

Regarding new options of treatment, the determination of the genetic status of the patient has proved to be an essential issue in the era of targeted therapies and personalized management strategies. An important aspect is to select those patients with the D816V KIT mutation who may respond to treatment with tyrosine kinase inhibitors (e.g., midostaurin and avapritinib), as well as identify those who have no *KIT* codon 816 mutations or exhibit other *KIT* mutations and may be sensitive to imatinib [57,95]. Nevertheless, tyrosine kinase inhibitors, which reduce MC burden, may be considered only in selected DCM patients with the most severe course of the disease who do not respond to intensive antimediator approaches [95]. In children with DCM, who usually display severe MC mediator-related symptoms, new drugs capable of reducing the pathogenic MC activity may turn out to be more effective and less toxic than MC depleters, such as tyrosine kinase inhibitors. MC activation antagonists, including omalizumab, for which only a few uncontrolled studies showing response in children with CM have been reported so far, or other monoclonal antibodies that engage inhibitory receptors (e.g., lirentelimab), might prove to be effective and become true therapeutic options in the near future [96,108,109]. Currently, early clinical trials of new drugs believed to reduce pathogenic MC activity are ongoing, which provides hope for patients with various MC disorders.

## Figures and Tables

**Table 1:** Updated Classification of Mastocytosis.

Cutaneous mastocytosis (CM) Maculopapular cutaneous mastocytosis (MPCM) ○ Monomorphic variant ○ Polymorphic variant Diffuse cutaneous mastocytosis (DCM) Cutaneous Mastocytoma
Systemic mastocytosis (SM) Non-advanced forms of SM Indolent systemic mastocytosis (ISM) Bone marrow mastocytosis (BMM) Smoldering systemic mastocytosis (SSM) Advanced forms of SM SM with an associated hematologic neoplasm (SM-AHN) Aggressive SM (ASM) Mast cell leukemia (MCL)
Mast cell sarcoma (MCS)

**Table 2:** A brief literature review of case reports on DCM.

	Disease Onset	Follow-Up Time	Anaphylaxis	Clinical Features and Outcome
Yasuda T. and Kukita A. 1962 [64]	At birth	3 months	NA	Hemorrhagic bullous lesions, diffuse brown pigmentation of the skin Episodes of severe fever and dyspnea No evidence of SM Death at 3 months of age
Allison J. 1967 [65]	At birth	8 days	NA	Generalized blistering, erythema Death at 8 days of age
Orkin et al., 1970 [63]	5 months	3 years	Once, 30 min after a meal	Generalized erythema, blistering, thickened skin No evidence of SM Partial improvement of skin lesions
Klaber M. et al., 1976 [66]	6 months and 2 months	25 years and 3 months	NA	Two cases Persistently thickened skin and pronounced Dermographism Erythematous, leathery skin
Harrison P. et al., 1979 [67]	20 days	4 years	NA	In the infantile period, erythema, blistering, and diffuse thickening of the skin with a leather-like appearance At the age of 4 years, there is slightly erythematous and thickened skin in some areas
Willemze R. et al., 1980 [60]	At birth	25 years	None	Persistent generalized erythema since birth Cutaneous tumors with MC infiltration on the arms and legs No evidence of SM
Olgun N. et al., 1993 [68]	5 months	NA	NA	Widespread bullous eruptions, small papules over the entire skin surface, and erythematous rash Intermittent flushing and fever; intense itching; MC infiltrates in BM After 7 months, some improvement
Shah P. et al., 1998 [39]	At birth	4 months	NA	Diffuse bullae Death at the age of 5 months due to bacterial endocarditis, pneumonia, and ascites
Murphy M. et al., 1999 [69]	At birth	17 months	NA	Generalized erythema, blistering Generalized lymphadenopathy Death at the age of 17 months
Enomoto U. et al., 1999 [70]	At birth	3 years	NA	Generalized brown, thickened leathery skin, and blistering No evidence of SM At the age of 36, there was slight blistering and hyperpigmentation
Waxtein L. et al., 2000 [71]	3 months	NA	NA	At the age of 3 months, generalized hyperpigmented skin with intense pruritus At the age of 10, diffuse infiltrated and hyperpigmented plaques on the trunk and extremities, and papular and yellowish nodules on the scalp and ears MC infiltrates BM
Tang X. et al., 2004 [53]	4 months	2 years	NA	Familial DCM with the A533D mutation Generalized pruritus of the skin and blistering on the scalp At the age of 2 years, the patient had only a solitary yellow-brown papule The patient’s father, paternal uncle, paternal grandfather, and paternal great aunt were diagnosed with DCM None of the family members had SM
Kinsler V. et al., 2005 [72]	10 weeks	6 years	NA	At the age of 10 weeks, there was an erythematous rash on the head, neck, and shoulders, thickened skin, and marked dermographism At the age of 6 years, small patches of urticaria
Walker T. et al., 2006 [73]	At birth	6 months	NA	Pachydermia, erythrodermia, dry skin and blisters
Duckworth A. et al., 2009 [74]	At birth	5 years	NA	Male fraternal twins Erythema, pink-brown macules and papules, diffusely thickened skin No systemic involvement Flushing, pruritus, and abdominal pain
Ghiasi M. et al., 2011 [75]	At birth	1 month	None	Generalized erythema, thickened skin, scattered nodules, and hemorrhagic bullae at birth Serum tryptase level: 6 ng/mL Hepatosplenomegaly Death at the age of 1 month due to an unknown reason
Kleewein K. et al., 2011 [28]	3 months	NA	NA	Generalized bullous eruptions and hemorrhagic crusts on the scalp, face, ear, and trunk Serum tryptase level: 58.9 ng/mL
Koga H. et al., 2011 [76]	At birth	14 months	None	Diffuse erythema and blisters on the scalp, face, and extremities At the age of 14 months, erythrodermic rash No evidence of SM.
Wawrzycki B. et al., 2013 [77]	At birth	7 months	NA	Tense blisters on thickened, erythematous skin on the face, trunk, and limbs No evidence of SM
Wang H. et al., 2014 [54]	5 months	35 years	NA	Familial DCM witch germline p.S451C mutation In the infantile period, generalized blistering At the age of 2 years, there is diffuse thickening of the skin and no blistering
Park M. et al., 2014 [62]	At birth	12 months	Once due to the unknown trigger	Diffuse leathery, erythrodermic rush, blisters on the head, neck, and trunk, with erosions on the face, scalp, and trunk Hepatosplenomegaly and mesenteric lymphadenopathy At the age of 12 months, hyperpigmented skin lesions
Otani I. et al., 2018 [36]	4 months	3 years	NA	Bullous skin lesions covering a third of the body surface, chest, and abdomen with almost complete reattached epidermis, back with healing, clean-based erosions Thickened skin with a yellowish color at the age of 5 months No further remission after 3 years of KIT K509I mutation Association with Tuberous Sclerosis
Hosking A. et al., 2018 [58]	6 months	NA	NA	Generalized bullous lesions Fevers and emesis No evidence of SM Partial improvement with time
Gupta M. et al., 2019 [78]	3 months	NA	NA	Blisters on the scalp and on the back Thickening of the skin on the abdomen and back No evidence of SM
Chaudhary N. et al., 2019 [35]	At birth	7 weeks	None	Generalized thickening of the skin with folds on the neck and inguinal regions, widespread hyperpigmented crusted lesions, some hypopigmented nodular lesions Hepatosplenomegaly (SM excluded) Death at 7 weeks of age due to acute respiratory distress and worsening cardiac function
Jenkinson H. et al., 2019 [40]	11 days	8 months	NA	Diffusely infiltrated red-brown skin with a marked leathery appearance and large, tense blisters on both hands No evidence of SM At the age of 8 months, some improvement
Li Y et al., 2020 [41]	At birth	NA	NA	Diffused red-brown papules and plaques on the scalp, face, trunk, and extremities Death at the age of one month due to severe infection, respiratory failure, and circulatory failure
Cardoso J. et al., 2020 [79]	At birth	NA	NA	Diffuse erythematous leathery plaques and tense bullaes No evidence of SM
Turnbull L. et al., 2020 [80]	At birth	NA	NA	Purpuric papules, macules, and bullae on the scalp, face, neck, chest, abdomen, and extremities No evidence of SM Some improvement with time
Rayinda T. et al., 2021 [81]	1.5 years	2 weeks	NA	Flaccid blisters on the face and body, papules, skin erosion, and erythematous wheals on the back of the face and chest
Wangberg H. et al., 2023 [52]	6 months	Ongoing	3 reactions provoked by peripheral intravenous placement and unknown triggers	Familial DCM with germline mutation A533D Diffuse blistering rash No evidence of SM At the age of 3 years, some improvement
Olteanu E. et. Al, 2023 [38]	At birth	2 years	NA	Thickened skin, generalized subcutaneous nodules on the face, scalp, trunk, back, hands, and feet At the age of 2 years, nodular lesions on the scalp and face

NA—not available.

**Table 3:** Skin diseases mimicking DCM.

Disease	Skin Lesions Resembling DCM	Main Clinical Features of the Disease
Staphylococcal scalded skin syndrome (SSSS)	Blistering Redness of the entire skin Desquamation of the skin	Denudation of the skin caused by exotoxin produced by phage group II strains of *Staphylococcus* species Usually presents 48 h after birth (rare in children older than six years) Culture from the site of the suspected primary infection is warranted
Epidermolysis bullosa (EB)	Generalized bullous eruptions	Genetic collagen disorder is characterized by skin fragility leading to blistering, wounds, and scarring Identification of typical gene mutations
Impetigo bullosa (IB)	Small vesicles that can grow into tense bullae and erosions	Superficial, highly contagious bacterial (*Staphylococcus aureus* and *Streptococcus pyogenes*) skin infection Pustules, blisters, and honey-colored crusted erosions Bacterial cultures can be used for confirmation of a diagnosis
Erythema multiforme (EM)	Blisters based on erythematous skin lesions	Target-like lesions present symmetrically on the extremities (especially on extensor surfaces) and spread centripetally Precipitating factors: infections, especially the herpes simplex virus, and medications Histology: vacuolar interface dermatitis with marked infiltration with lymphocytes along the dermo-epidermal junction
Atopic dermatitis	Pruritic rash, erythroderma in severe cases	A defect in the skin barrier causes xerosis. Severe pruritus In infants, edematous papules and plaques that may have vesicles or crust on the scalp, face, and extensor extremities
Langerhans cell histiocytosis	Extensive rush and blistering in infants	Clonal disease of the monocyte-macrophage system A wide spectrum of skin lesions Histology with immunophenotyping: accumulation of CD1a-positive and/or CD207-positive dendritic cells
Linear IgA bullous dermatosis	Plaques and papules with blistering	Widespread annular blisters that exhibit a predilection for the lower abdomen, thighs, and groin Direct immunofluorescence: linear IgA deposits on the basement membrane zone
Incontinentia pigment	Blistering rash	Blistering, present in the early stages of infancy, heals spontaneously Blistering stage, followed by the development of verrucous lesions verrucous lesions and hyperpigmentation Coexisting signs: hair loss (alopecia) and dental abnormalities

## Data Availability

Not applicable.
